# Plant exosomes: nano conveyors of pathogen resistance

**DOI:** 10.1186/s11671-023-03931-4

**Published:** 2023-11-30

**Authors:** D. Subha, R. AnuKiruthika, Harsha Sreeraj, K. S. Tamilselvi

**Affiliations:** 1 Department of Biotechnology, PSGR Krishnammal College for Women, Coimbatore, India; 2 Department of Botany, PSGR Krishnammal College for Women, Coimbatore, India

**Keywords:** Exosomes, Extracellular vesicles, Cross-kingdom, Host–pathogen, SIGS

## Abstract

The entry of a pathogen into a plant host is a complex process involving multiple steps. Survival techniques from the pathogen and the defense mechanisms of the plant lead to a plethora of molecular interactions during the operation. Plant extracellular vesicles, especially the exosomes in the size range of 50–150 nm play a crucial role in plant defense. They act as signalosomes capable of transporting bioactive lipids, proteins, RNA and metabolites between the host and the pathogen. Recent research works have revealed that anti-microbial compounds, stress response proteins and small RNA are among the contents of these extracellular vesicles. The current review article analyses the cruciality of the cross-talk between the host and the pathogen organized through trafficking of small RNA via exosomes towards RNA induced gene silencing in the pathogenic organisms. Recent studies have shown that extracellular vesicles released by both plants and the pathogens, play a crucial role in cross-kingdom communication, thereby regulating the host response and contributing to plant immunity. An in-depth understanding of the mechanism by which the EVs mediate this inter-species and cross-kingdom regulation is currently needed to develop sustainable plant-protection strategies. The review highlights on the latest advances in understanding the role of EVs in establishing host–pathogen relationship, modulating plant immunity and approaches for how these findings can be developed into innovative strategies for crop protection.

## Introduction

Host–pathogen relationship involves molecular interactions at various levels and also transfer of biologically active molecules between themselves. During the course of an infection, the pathogen tries to establish entry and colonization by expressing various virulence genes, whereas the plant uses all possible strategies to ward off and kill the pathogen. As the most crucial part of the infection, the entry of the pathogen, involves interactions in the intercellular spaces, a lot secreted molecules play a role in the process. The means of their transfer and delivery to the right space still remains unclear. Extracellular vesicles (EVs) are heterogenous phospholipid bilayer membrane bound spherical structures that carry biologically active cargo including lipids, proteins and nucleic acids. They have been implicated in cell to cell communication and transfer of biomolecules [1, 2]. Currently, EVs are categorized based on their origin and their size. The different types of extracellular vesicles include microvesicles, exosomes and apoptosis-derived vesicles. Exosomes are nanosized (50–150 nm) bodies held within the intraluminal vesicles of multi-vesicular bodies and are released by their binding with the plasma membrane [3, 4]. Microvesicles are comparatively larger nanostructures (100 nm -1000 nm) produced by the direct budding of plasma membrane. Apoptosis-derived vesicles are larger structures produced during apoptosis, when membrane blebs-off of the dead cells. There are several recent reports that state the importance of extracellular vesicles in achieving the transport of various plant-defense and virulence factors between the plant and the pathogen [2, 5–8]. Understanding the relationship between the host and the pathogen is important to devise effective pathogen-control measures. So there is a significant rise in the research interest on the extracellular vesicles, which play the role of cross-kingdom communicators.

## Anti-microbial compounds in plant EVs

Plants produce more EVs in response to biotic and abiotic stresses. Infection with *Pseudomonas syringae* and treatment with salicylic acid induces increased secretion of exosomes [9]. It is also known that the MVBs proliferate when the plant undergoes stress [10]. It can be considered as a general immune response because these EVs are enriched with antimicrobials and stress-related proteins. Gene ontology studies on the proteome of EVs obtained from the *Arabidopsis* leaves revealed that about 26% of the proteome falls under the category of proteins produced in response to biotic and abiotic stimuli. The proteome involved signal transmission proteins that contribute to immune response such as RPM1-INTERACTING PROTEIN4 (RIN4), which is capable of interacting with bacterial effectors and triggering immunity. The enrichment of proteins involved in immune signaling suggests that the exosomes might play a role in spreading signals to the cells to activate pathogen detection. Defense related proteins involved in myrosinase-glycosinolate system such as PEN3, NRT1 and the myrosinase epithiospecific modifier 1 and ROS signaling proteins were also identified in the EV proteome [11, 12]. Interestingly many pathogenesis related (PR) proteins such as chitinase II, thaumatins, proteinase inhibitors, peroxidases and lipid transfer proteins were identified in the proteome of the EVs extracted from the sunflower seedlings. It was found that the spores of the phytopathogenic fungus could internalize the purified EVs produced by the seedlings and moreover, the EVs reduced the viability of the spores and also affected hyphal growth in the germinating spores clearly demonstrating an anti-fungal effect [13].

When the proteins extracted from the EVs isolated from the root exudates of tomato were analyzed, a high proportion of them were found to be involved in plant defense. Proteins involved in immune responses such as late blight resistance proteins RIA-3,10 and two endochitinases capable of decomposing pathogen cell wall were identified. In line with that, these EVs significantly inhibited the spore-germination and the development of germination tubes in the plant pathogens *Fusarium oxysporum, Botrytis cinerea* and *Alternatis alternata* [14].

*Arabidopsis* PEN gene products are known to accumulate at the point of entry of the fungi. They act at the cell periphery and execute the apoplastic immune responses to limit fungal entry. It is believed that the transport of these defense related proteins normally associated with plasma membrane, to the pathogen induced extracellular spaces occurs through the biogenesis and release of the exosomes [15]. In Barley leaves, as a response to powdery mildew fungus extracellular vesicles accumulate at the region of pathogen entry, and deposit signaling components, calcium, elicitors and antimicrobial phenolics and hydrogen peroxide at the site [16]. *Arabidopsis *PEN3 accumulates at the sites of attempted penetration in response to molecular cues from the pathogen such as flagellin and chitin. The beta-glucosyl hydrolase PEN2 and the ABC transporter PEN3 play a role in a pathway to transport antimicrobial compounds across the plasma membrane into the extrahaustorial matrix that surround and delimit the haustoria of the powdery mildew fungi [17]. Interestingly these proteins lack the usual signal peptides required for localization, suggesting the existence of a unique pathway for the focal accumulation of the effectors [9, 18]. Reinforcement of the cell wall occurs through deposition of cell wall appositions at the site of pathogen entry forming unique structures called papillae. Reports suggest that MVBs accumulate during infection and the secreted vesicles are locally concentrated and embedded in the papillae [19]. Increased enrichment of extracellular vesicles has also been observed in the extrahaustorial matrix, the space at the interface of the plant cell and the invading fungal haustorium [20].

From the above reports it can be inferred that the role of plant extracellular vesicles in plant defense is multifaceted (Table 1). They function as mobile pockets highly enriched with antimicrobials and plant-defense proteins which initiate the plant immune response at the site of pathogen entry. They are concentrated with signaling compounds and might be involved in transferring signals to the cells about pathogen recognition. The biogenesis and release of vesicles even in the uninduced plant systems as in the tomato roots, indicate that they might be released to form a protective barrier of plant defense compounds around the parts vulnerable to pathogen entry such as wounded roots.

**Table 1:** List of plant-pathogen systems in which exosomes play a role in plant-defense

Plant system used for testing	Pathogen	Isolation	Role	References
*Arabidopsis thaliana*	*Acidovorax citrulli* *M6*	Centrifugation; filtration; ultracentrifugation; Optiprep	Activation of defense-related gene expression	[5]
*Arabidopsis thaliana*	*Xanthomonas campestris pv campestris 33913*	Centrifugation; filtration; ultracentrifugation; Optiprep	EV-induced ROS burst, activation of defense-related gene expression	[5]
*Arabidopsis thaliana*	*Xanthomonas oryzae pv oryzae PXO99*	Centrifugation; filtration; ultracentrifugation; Optiprep	Activation of defense-related gene expression	[5]
*Arabidopsis thaliana*	*Pseudomonas syringae pv tomato DC3000*	Centrifugation; filtration; ultracentrifugation; Optiprep	Activation of defense-related gene expression	[5]
*Arabidopsis thaliana*	*Pseudomonas syringae DC3000*	Apoplastic fluid extraction; filtration; ultracentrifugation; Optiprep	Increased EV production in response to *Pseudomonas syringae DC3000* and salicylic acid; EV proteome enriched in plant defense components	[11]
*Arabidopsis thaliana*	*Botrytis cinerea*	Apoplastic fluid extraction; filtration; ultracentrifugation	TET8-GFP-labeled *Arabidopsis* EV accumulation around the infection site; EV-mediated sRNA uptake by *Botrytis cinerea* cells to silence pathogen virulence-related genes	[47]
*Hordeum vulgare*	*Blumeria graminis f sp. Hordei*	–	Enhanced vesicle accumulation around haustoria in barley leaf attacked by fungal pathogen *Blumeria graminis* f.sp. hordei; vesicle releasement into paramural space via plant-derived MVB fusion with plasma membrane	[31]
*Helianthus annuus L*	*Sclerotinia sclerotiorum*	Extracellular fluid extraction; filtration; ultracentrifugation	Antifungal activity against *Sclerotinia sclerotiorum* ascospores: treatment with sunflower-derived EVs resulted in inhibited pathogen growth and/ or cell death; EV proteome enriched in plant defense components against pathogen	[13]
*Helianthus annuus L*	*Golovinomyces cichoracearum*	Differential centrifugation	Antifungal activity, enriched in cell wall enzymes	[13, 48]
*Gossypium hirsutum*	*F. oxysporum f. sp. vasinfectum*	Centrifugation; filtration; ultracentrifugation; Optiprep	The extracellular vesicles of the pathogen cause phytotoxic response in plants	[46]

## Extracellular vesicle mediated cross-kingdom RNAi

**Fig. 1:**
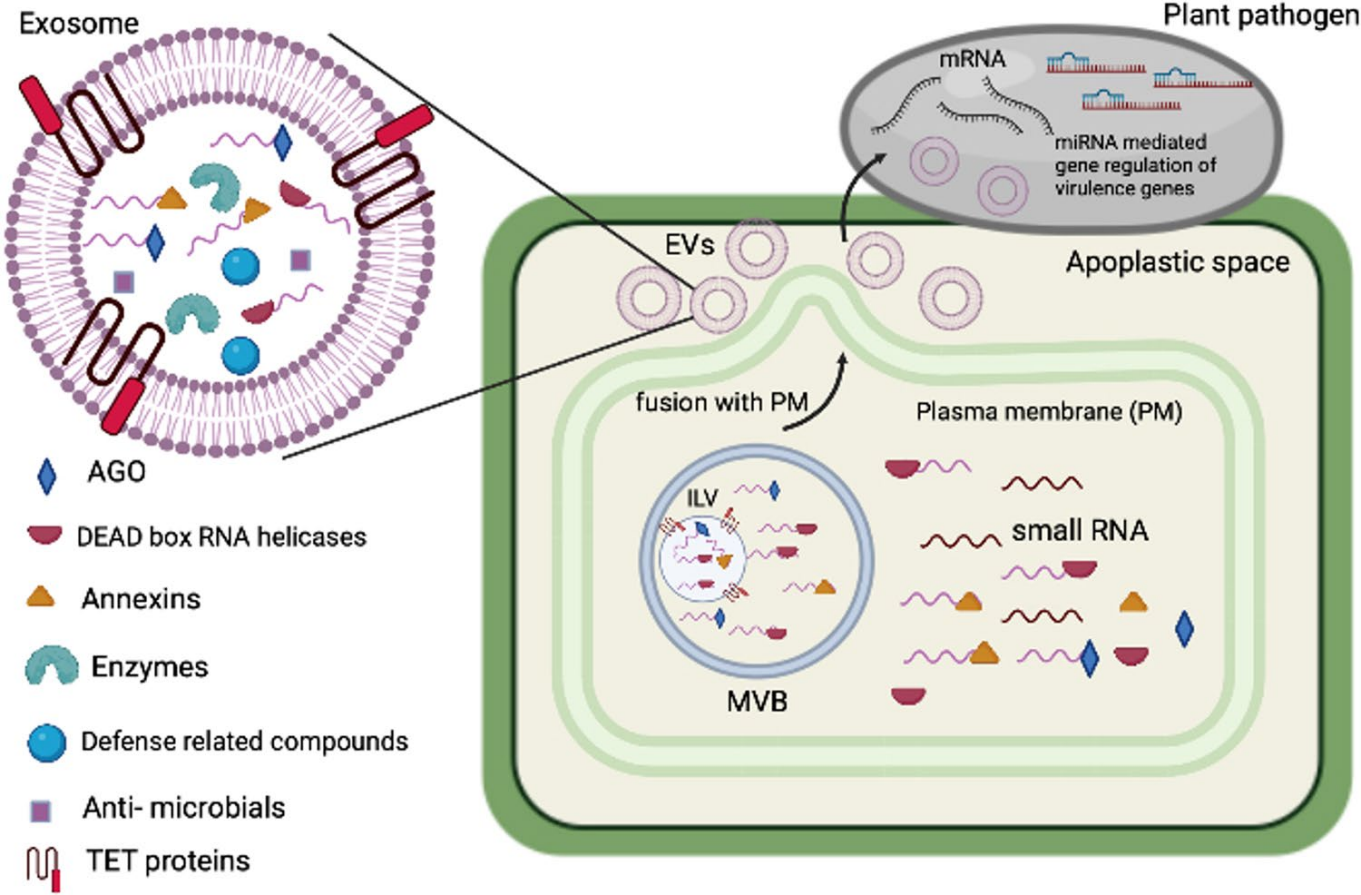
Extracellular vesicle mediated cross-kingdom RNAi. Schematic representation of the role of plant exosomes, which act as ‘signalosomes’ with their unique content as shown, in the protection of plant from the pathogens

Multiple molecular interactions between the host and the pathogen occur during an infection. Small RNAs have a crucial place as effectors that are transferred between them, from the pest to suppress host immunity and from the host to inhibit their virulence (Fig. 1). Small RNAs are non-coding RNAs of size range 19–25 nt which are generated and used for gene silencing throughout Eukaryota. Even though sRNA trafficking between cell to cell within plants has been well studied, their mode of transmission between host and pathogen still remains unclear.

Wang et al., have demonstrated for the first time that the RNAi existing between the pathogen and the host is bidirectional [21]. The same group has previously shown that *Botrytis cinerea* delivers small RNAs into the plant cells that weaken the immune response [22]. Interestingly, expressing sRNAs that target Bc-DCL1 and Bc-DCL2 in *Arabidopsis* and tomato led to the silencing of Botrytis -DCL genes which are crucial for synthesis of small RNA, thereby attenuating fungal pathogenicity [21]. The ability of the fungal pathogen to uptake external RNAs have been clearly demonstrated, implicating bidirectional nature of cross kingdom RNAi and small RNA trafficking between the fungal pathogen and plant host. The same has already been established in animal systems [23]. Taken together, it can be implied that the cross kingdom RNAi has evolved in both plant and animal systems as a conserved virulence mechanism. The first report of transfer of plant miRNA into fungal pathogen came from Zhang et al., 2016 [24]. In response to infection with *Verticillium dahlia*, cotton plants transfer their endogenous miRNAs miR156 and 159 to the pathogen. These miRNA target the fungal virulence genes Ca2 + dependent cysteine protease and isotrichodermin C-15 hydrolase respectively.Cai et al., used a sequential protoplast isolation method to study the difference in the concentration of endogenously transferred plant small RNA between the fungal cells and plant cells. The findings indicate that the host cells transferring the small RNA into the pathogens occurs in a regulated highly selective process [25]. Extracellular vesicles have already been implicated in transfer of small RNAs in animals. *Heligmosomoides polygyrus*, which is a native mouse intestinal parasite, uses exosomes for trafficking of the miRNAs into the intestinal macrophages to suppress host immunity [23]. In line with that, when the vesicular small RNAs isolated from the apoplastic fluid of the infected leaves were compared with the sRNAs transferred to the fungal cells, it was found that approximately 74% of them were present in the vesicles. Moreover, the small RNAs were able to survive nuclease treatment suggesting protection from the surroundings during transport. Tetraspanin 8 (TET 8) is similar to CD63, a specific mammalian marker for exosomes. TET8-GFP labelled exosomes were taken up efficiently by the fungal cells within 2 h along with its cargo of small RNAs. This observation suggests that exosomes function to transfer small RNA into fungal cells [25].

## Selective loading of small RNAs into extracellular vesicles

Previous investigations suggest that the loading of small RNAs into the extracellular vesicle is not a random process, but a tightly regulated specific one. But the machinery and the process involved in this selective loading of cargo into the extracellular vesicles is still not clear. When the proteome of the plant EVs from *Arabidopsis* was analyzed, a total of 93 RNA binding proteins were recognized. Of which the important ones with ssRNA binding capacity are AGO1, DEAD-box RNA helicase RH11, RH37, RH52, Annexin 1 and ANN2. These proteins were also easily detected by western blotting in isolated EVs thereby confirming the proteome results. These RBPs co-localize with the TET8 marker and the EV associated small RNAs previously identified (such as TAS1c-SiR483, TAS2-SiR 453 and miR166) were also found associated with this subgroup of exosome like EVs. EV localized RBPs specifically bind with the small RNAs and aid in their selective loading into the EVs. The RBPs, AGO1 and RH37 can only selectively bind with the EV-enriched small RNAs in the total cell extraction, indicating a role for these proteins in binding a specific set of sRNAs and carrying them into EVs for secretion. The Annexins (ANN1 and ANN2) which were also found in the EVs do not bind with sRNAs specifically and might contribute to the stabilization of the RNA inside the vesicles. The mutants of these RBPs *rh1rh37* and *ann1ann2 *were more susceptible to *B.cinerea* infection and they couldn’t generate exosomes with specific small RNAs against the pathogen [26].

## Uptake of extracellular vesicles

The communication between the plant host and the fungal pathogens via extracellular vesicles is intriguing because of the requirement to cross dual cell wall barriers. Direct uptake of EVs by fungal cells have been demonstrated [25]. But the exact mechanism that dictates the crossing of cell wall and release of effectors is not clear. In mammalian system, the uptake of EVs can occur through one of the four ways namely (i) phagocytosis (ii) micropinocytosis (iii) caveolin mediated endocytosis or by (iv) direct fusion at the target cell plasma membrane [27]. As the plant and the fungal systems lack caveolin or phagocytosis, clathrin mediated endocytosis or direct fusion are the possible methods for the uptake of EVs. Clathrin independent pathways are also known to occur in plants. Remorin 1.3, a marker for clathrin independent endocytosis accumulates in the haustoria of *Phytophthora infestens*. It was found to co-localize with pathogen effectors thereby increasing the susceptibility of infection [28]. Moreover remorin is implicated in the regulation of plasma membrane topology and scaffold formation [29], suggesting that it could modify the pore-size, elasticity or other physical characteristics to enhance the traffic of EVs. In addition to the membrane proteins, the EVs can also hold cell wall remodeling enzymes. On sensing an infection, the plant tries to strengthen the defense by improving the cell wall integrity, while the pathogen takes advantage of the cell wall metabolism in the host to establish the infection [30]. These cell wall remodeling proteins are transported through EVs in several cases. The proteomic studies on EVs from sunflower seedlings revealed that 47% of the identified proteins are cell wall associated including the enzymes in-charge for polysaccharide reorganization [13]. These studies clearly indicate that the EVs are quite capable of modulating the cell wall barriers with their cargo.

## EV based methods for crop protection

**Fig. 2:**
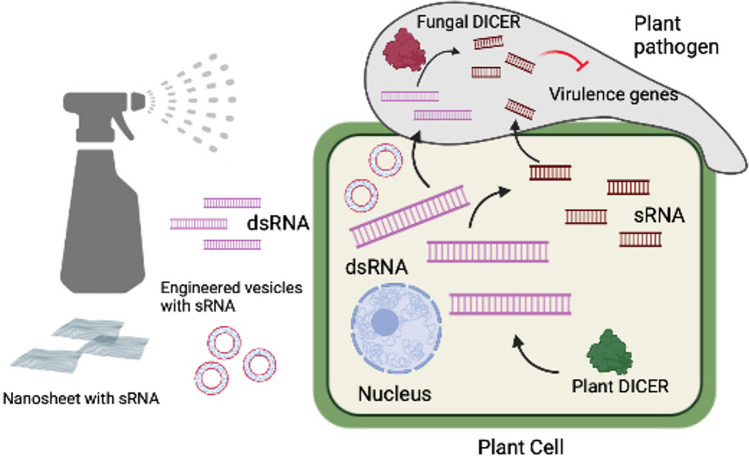
Spray induced gene silencing (SIGS). Schematic depiction of cross kingdom RNAi based Spray Induced Gene Silencing (SIGS), a promising crop protection strategy against pathogens

Cross kingdom RNAi has been exploited to develop pathogen resistance through host-induced gene silencing (HIGS) [31]. This is achieved by transforming plant with the double stranded RNA construct targeting the virulence genes in the pathogens. The dsRNAs and siRNA produced in the plant finds entry into the pathogen and silences the virulence genes. The main limitation of this process is the requirement to develop transgenics. With the interesting finding that spraying of dsRNA and sRNA targeting the pathogen genes, on the surface of the crops can successfully inhibit pathogenesis, a new strategy for disease control termed spray induced gene silencing (SIGS) was devised [21, 32] (Fig. 2). For example [33], have demonstrated that spraying of dsRNACYP3, that targets three cytochrome p450 lanosterol C14-alpha demethylases inhibits the growth of *Fusarium germinearum* on barley. Spraying of dsRNA targeting BcDCL1 and DCL2 on the fruits and vegetables significantly reduced the infection with *B.cereus* [21]. Similar application of dsRNA protects *Brassica napus* from *S.sclerotiorum* and *B.cereus* [34]. The basic concept behind SIGS is the environmental RNAi or the ability of the organism to uptake the RNAs from the surroundings. The effectiveness of the strategy depends upon the RNA stability and uptake efficiency of the pathogens. However, a conclusive evidence for the involvement of EVs in transfer of dsRNA in SIGS and the mechanism behind the transfer still remains lacking [35]. The fact that RNA get easily degraded upon exposure to various environmental conditions is a huge limiting factor for the applications of SIGS. The advancement of these crop protection strategies require safe and efficient carriers for the sRNA to be developed. Extracellular vesicles are widely used by plants [36] and animals in nature to transport RNA without degradation. Efforts have been made to design artificial nanovesicles mimicking EVs to improve the stability and internalization of RNA in SIGS. Recently, artificial nanovesicles synthesized using cationic lipid formulations were tested for dsRNA delivery and uptake by *B.cinerea*. The Avs provided a strong shielding to the enclosed RNA and the duration of the protection conferred by the RNA in this case, was significantly improved. In another strategy, anchoring the pathogen specific dsRNA on layered double hydroxide (LDH) clay nanosheets before spraying significantly improved the stability of the RNA and provided prolonged resistance [37]. Liposomes composed of plant-derived lipids, when loaded with agricultural nutrients and applied on the surface of tomato leaves were able to penetrate the leaves and deliver them [38–40]. There are several reports on the usage of liposomes for the delivery of nucleic acids to CRISPR constructs [38–40]. Numerous similarities exist between the liposomes and exosomes, suggesting that they can also be utilized for targeted delivery of macromolecules and nucleic acid. Since EVs are implicated in the transport of sRNAs from plants into their fungal pathogens, incorporating the RNAs into EVs or engineered exosomes can enhance its stability and delivery via SIGS. Research on application of liposome engineering technologies to modify exosomes to enclose and deliver sRNA is underway.

The Outer membrane vesicles (OMVs) which are released by the gram negative bacteria from the outer membrane into the extracellular milieu, plays a significant role in establishing the host–pathogen interaction. The immunomodulatory role of OMVs has been well recognized in mammalian systems and has been harnessed for vaccine development. However, the research on the interaction between the OMVs from plant pathogens and plant systems still remain rudimentary. Recently, McMillan et al., have shown that OMVs from bacteria can efficiently elicit plant immune response. Exposure to OMVs from *Pseudomonas syringae* and *Pseudomonas fluorescens* can mount a broad spectrum immune response against bacterial and oomycete pathogens [41]. By activating various immune pathways, a complex immune response is induced by the species specific immune elicitors present in the OMVs. Interestingly, the local immunity elicited by the OMVs from the commensals can lead to systemic immune protection against pathogens in plants [42]. It has been shown that OMVs from *Xanthomonas campestris* pv. *Campestris* directly interacts with the *Arabidopsis thaliana* plasma membrane in a host nanodomain and Remorin dependent manner thereby altering its properties and inducing innate immune response [43]. A study on transcriptomic changes between *Xanthomonas campestris* OMV treated and wildtype *A.thaliana* seedlings reveal a major transcriptional shift towards immune activation with upregulation of multiple immune receptors and modulators [44]. Understanding the immunomodulatory effect and the inter-kingdom communication mediated by OMVs can lead to better disease management strategies in agricultural crops.

As EVs are produced by plants, commensals and pathogens, an emerging perspective of microbiota EVs (MbEVs) has been gathering considerable research attention [45]. EVs, being significant carriers of bioactive cargo between cells, they play a critical role in defining the relationship between microbiota and plant host cells. MbEVs also carry some immunogenic cargos in addition to those involved in plant–microbe interactions, which provides them a potential to aid in a variety of plant defense mechanisms. The microbiota associated with the plant influences the colonization of microbial pathogens and also the bioactive signals from their EVs modulates the plant immune response. The prime function of the microbiome is to minimize the risk of invasion by the pathogens and insect pests. The exogenous application of MbEVs can inhibit the growth of phytopathogens and elicit an immune response. The plant derived and the microbiota derived EVs remain in a sync to provide the optimal immunity to the plant against the pathogens. This contributes to a difference in the MbEVs between the healthy and infected parts of the plant as well as between the susceptible and resistant plant varieties. Consequently, MbEVs could be employed as biomarker for plant diseases. Elucidating the EV link between the plants and their associated microbiome can lead to effective plant protection strategies without the involvement of harmful chemicals.

## Future perspectives and challenges

Recent research works clearly demonstrate that EVs are mobile signalosomes which play a crucial role in communication and signaling not only between the cells of the plant but also between the plant host and the pathogen. Their role extends from recognition of pathogen effectors to trafficking of antimicrobial metabolites, delivery of small RNAs that target pathogen genes, forming a physical barrier towards pathogen infiltration, modulation of Pattern Recognition Receptor (PRR) activity and transfer of distress signals. This property of EVs in plant immunity is translated into plant defense strategies such as SIGS. However, many aspects related to the process remains unknown. Understanding the host–pathogen relationship at molecular level would help in designing non-transgenic anti-pathogen measures and sustainable agricultural practices. Many advantages associated with mammalian EVs is still lacking with the research on plant EVs. The surface markers on the extracellular vesicles to differentiate between their types are yet to be identified. A database for plant EV proteins and small RNAs is not available for quick identification. The clear mechanism underlying the trafficking of the EVs is unknown. The question of whether the process is specific and cargo dependent needs to be answered. Trans kingdom RNAi needs to be further decoded to understand its role in plant immunity. The question of whether the levels of small RNA identified in the pathogens is enough to cause a physiological change still remains. The possible additional roles for these small RNAs such as epigenetic consequences need attention. The small RNAs transferred to the pathogens from the plants might execute a specific-gene mediated regulatory pathway or be a part of a complex regulatory network. With highly evolved modern next-generation sequencing techniques available now, many novel small RNAs from different sources are identified. Experimental confirmation of ability to silence genes in a trans-kingdom fashion is pending in many such cases. Comparative genomics and deep sequencing microbiome projects are need of the hour to identify more effective cross-kingdom targets that can be used for development of SIGS.

## Conclusion

The extracellular vesicles secreted by both plants and microbes, as novel carriers of biologically functional cargo, mediate a delicate interchange of information. This EV-mediated communication is critical for the survival of both plants and micro-organisms. Research on the role of EVs in plant-pathogen interaction is still in its infancy. With newly found developments in the field such as microbiotic EVs and OMVs from bacterial pathogens, the realization that EVs hold an indispensable role in mediating microbial pathogenesis, virulence, microbial competition and plant immunity has dawned on the scientific community. Efforts towards understanding the mechanistic basis of delivery and interaction of RNA containing extracellular vesicles can lead to innovative RNAi based plant protection systems. The development of novel concepts like using of OMVs and mbEVs as vaccine against plant infections and EV transplants for microbiome manipulation reliably forecasts the indispensable role for EVs in crop management in future. A meticulous research on the biology of EVs and translation of the obtained knowledge might provide a colossal opportunity to develop sustainable and eco-friendlier plant protection systems.

## Data Availability

Not applicable.
